# Counterproductive effects of anti-CD38 and checkpoint inhibitor for the treatment of NK/T cell lymphoma

**DOI:** 10.3389/fimmu.2024.1346178

**Published:** 2024-04-12

**Authors:** Wendy W. L. Lee, Jing Quan Lim, Tiffany P. L. Tang, Daryl Tan, Ser Mei Koh, Kia Joo Puan, Liang Wei Wang, Jackwee Lim, Kim Peng Tan, Wee Joo Chng, Soon Thye Lim, Choon Kiat Ong, Olaf Rotzschke

**Affiliations:** ^1^ Singapore Immunology Network (SIgN), Agency for Science, Technology and Research (ASTAR), Singapore, Singapore; ^2^ Lymphoma Translational Research Laboratory, Division of Cellular and Molecular Research, National Cancer Centre Singapore, Singapore, Singapore; ^3^ Oncology-Academic Clinical Programme (ONCO-ACP), Duke-National University of Singapore (NUS) Medical School, Singapore, Singapore; ^4^ Division of Medical Oncology, National Cancer Centre Singapore, Singapore, Singapore; ^5^ Clinic for Lymphoma, Myeloma and Blood Disorders, Mount Elizabeth Hospital Novena Specialist Centre, Singapore, Singapore; ^6^ Cancer Science Institute of Singapore, National University of Singapore, Singapore, Singapore; ^7^ Department of Medicine, Yong Loo Lin School of Medicine, National University of Singapore, Singapore, Singapore; ^8^ Department of Hematology-Oncology, National University Cancer Institute of Singapore, National University Health System, Singapore, Singapore; ^9^ Director’s Office, National Cancer Centre Singapore, Singapore, Singapore; ^10^ Office of Education, Duke-National University of Singapore (NUS) Medical School, Singapore, Singapore; ^11^ Cancer and Stem Cell Biology, Duke-National University of Singapore (NUS) Graduate Medical School, Singapore, Singapore; ^12^ School of Biological Sciences, Nanyang Technological University, Singapore, Singapore

**Keywords:** immunotherapy, lymphoma, T cell activation, checkpoint inhibition, combination therapy

## Abstract

**Introduction:**

Natural killer/T cell lymphoma (NKTL) is an aggressive malignancy associated with poor prognosis. This is largely due to limited treatment options, especially for relapsed patients. Immunotherapies like immune checkpoint inhibitors (ICI) and anti-CD38 therapies have shown promising but variable clinical efficacies. Combining these therapies has been suggested to enhance efficacy.

**Methods:**

We conducted a case study on a relapsed NKTL patient treated sequentially with anti-CD38 followed by ICI (anti-PD1) using cytometry analyses.

**Results and Discussion:**

Our analysis showed an expected depletion of peripheral CD38+ B cells following anti-CD38 treatment. Further analysis indicated that circulating anti-CD38 retained their function for up to 13 weeks post-administration. Anti-PD1 treatment triggered re-activation and upregulation of CD38 on the T cells. Consequently, these anti-PD1-activated T cells were depleted by residual circulating anti-CD38, rendering the ICI treatment ineffective. Finally, a meta-analysis confirmed this counterproductive effect, showing a reduced efficacy in patients undergoing combination therapy. In conclusion, our findings demonstrate that sequential anti-CD38 followed by anti-PD1 therapy leads to a counterproductive outcome in NKTL patients. This suggests that the treatment sequence is antithetic and warrants re-evaluation for optimizing cancer immunotherapy strategies.

## 1 Introduction

NK/T-cell lymphoma (NKTL) is a rare and aggressive form of an Epstein Barr Virus (EBV)-associated cancer with a predilection for Asian and South American populations (1). Early stage NKTL patients are typically treated with a combination of radiotherapy and chemotherapy, while L-asparaginase based regimens such as SMILE (steroid, methotrexate, ifosfamide, L-asparaginase and etoposide) are given at late stages or relapsed patients (2). However, these chemotherapy regimens typically give rise to adverse events such as high grade lymphopenia and increased infection risks, leaving SMILE refractory NKTL patients with a poor prognosis (3).

In recent years, advances in immunotherapy provide a new avenue for the treatment of cancer patients. By harnessing the body’s natural defense to fight cancer cells, this approach has proven to be effective for the treatment of many cancers (4–8). One of these strategies is to destroy cancer cells by injecting depleting antibodies directed against tumor-specific surface markers. Daratumumab (anti-CD38) was found to effectively deplete multiple myeloma (MM) cells as these cells express high levels of CD38 (9). A case report showed that NKTL tumors also express CD38 and suggested that Daratumumab monotherapy may be efficacious in NKTL patients (10). A recent clinical trial however reported only limited clinical benefit for NKTL patients, casting some doubt on the usefulness of Daratumumab for this type of cancer (10, 11). More importantly, CD38 expression is not restricted to the transformed tissue. It is present on various immune subsets including B cells (12) and, as a common activation marker on NK cells (13, 14) and T cells (15–17). Thus, we hypothesize that the use of Daratumumab may thus also have some compromising effect on the immune system.

Another promising approach appears to be immune checkpoint inhibition (ICI). ICI therapies disrupt the sensing of inhibitory receptor signals delivered from the transformed tissue by tumor-specific T or NK cells to reinvigorate their cytotoxic capacity (18). Currently the most promising target is the PD1/PDL1 axis. PD1 is an immunoinhibitory molecule expressed on activated lymphocytes including CD8+ T cells (19). The ligand (PDL1) is commonly found on antigen-presenting cells and often expressed by tumor cells (20, 21). Ligation of PD1 with PDL1 inhibits T cell cytotoxicity (21, 22) and the use of anti-PD1 perturbing this receptor/ligand interaction can unleash the suppressed cytotoxicity of T cells to kill the tumor cells (23). In patients with relapsed/refractory NKTL the efficacy of ICI therapy was demonstrated with anti-PD1 antibodies such as Pembrolizumab or Nivolumab, which reportedly achieved complete response (CR) in around 30-70% of the patients (24–28).

In spite of the apparent effectiveness of ICI-based immunotherapies, they often work only in a subset of patients (24–27). A common strategy to increase treatment efficiency is through the combination with other (immune-) therapies to synergize their effects (29). For NKTL, it had been suggested that the combined use of anti-PD1 and anti-CD38 therapy may help to improve the low efficacy rates of these monotherapies (30). However, here we show that a combination of these two treatments is in fact counterproductive: the residual depleting anti-CD38 antibody (Daratumumab) effectively eliminates the effector CD8+ T cell populations that were (re-)activated by the anti-PD1 treatment and was associated with a strong upregulation of CD38. The counteracting effect of Daratumumab on checkpoint inhibition was evident more than 6 weeks after completing Daratumumab therapy. Residual levels of Daratumumab were still sufficient to deplete all effector memory CD8+ T cells, typically induced by the anti-PD1 treatment.

## 2 Materials and methods

### 2.1 Ethics approval and consent to participate

Fresh blood samples were obtained from healthy volunteers and patients in accordance to the Helsinki declaration. Healthy donor samples were collected under the SingHealth Centralised Institutional Review Board (CIRB Ref: 2017/2806) and HSA “Residual Blood Samples for Research” project titled “Blood biomarkers of immune-related diseases” (Ref: #201306-05). Patient samples were obtained under SingHealth CIRB Ref: 2004/407/F. Written informed consent was obtained from all donors prior to sample collection.

### 2.2 Search strategy and selection criteria for meta-analysis

The databases used to retrieve relevant articles include National Center for Biotechnology Information (NCBI) Pubmed and ClinicalTrials.gov. The keywords used are “anti-CD38 therapy, anti-PD1 therapy, anti-PDL1 therapy, combination therapy trials, cancer trials, Daratumumab, Pembrolizumab and Nivolumab”. All studies with cancer patient receiving either anti-PDL1/anti-PD1 alone or in combination with anti-CD38 were included. Subsequently, trials in which patients received anti-CD38 or ICI in combination with another agent were excluded. Incomplete trials or trials with insufficient information were also not included. Anti-CD38 monotherapy studies which consists of only multiple myeloma patients were excluded from analysis since CD38 acts directly on the myeloma cells and not within the scope of this study (9). The Preferred Reporting Items for Systemic Reviews and Meta-Analyses (PRISMA) flow diagram can be found in **Supplementary Figure 6**.

### 2.3 Quantitation of genomic EBV DNA levels in patient plasma samples

Patient plasma samples were split into two fractions and treated either with Buffer RDD (Qiagen) or DNAse I (Qiagen). DNA detected from the DNAse I-treated fraction represents only virion-encapsidated DNA, while mock-treated DNA (by Buffer RDD) comprises the total DNA in the patient’s plasma (cell-free host DNA, virion-encapsidated DNA and free-floating viral DNA). An equal volume of cold phenol-saturated Tris-EDTA (10 mM Tris, pH 8.0 with 1 mM EDTA) was added to each tube followed by vigorous shaking. Samples were then centrifuged at 13,000 relative centrifugal force (rcf) at 4°C for 15 mins. An additional 100 μL of Buffer EB (Qiagen) was gently added to facilitate aspiration. The aqueous phase of DNA was extracted and stored at -20°C until use. Standard ethanol precipitation using glycogen as carrier was performed to concentrate the DNA. Buffer EB (Qiagen) was used to resuspend the purified DNA. Virus DNA quantification was performed in accordance to published qPCR protocol using 1 μL template DNA (31). Standard curve was generated using DNA from the Namalwa EBV-positive Burkitt’s lymphoma cell line (32).

### 2.4 Anti-EBV viral capsid antigen IgG and total IgG quantification

Quantification of total IgG was performed according to manufacturer’s protocol using Human IgG ELISA quantification set (Bethyl Laboratories) with heat inactivated human plasma samples diluted 1:50,000. Quantification of anti-EBV VCA IgG was performed according to manufacturer’s protocol using EBV-VCA IgG ELISA kit (Calbiotech) using heat inactivated human plasma samples.

### 2.5 Competitive anti-CD38 staining

NK92 cells were first stained using the Fixable Aqua Dead Cell Kit (Thermofisher), followed by incubation with either with titrated amounts of Darzalex (Daratumumab) (Johnson & Johnson), or plasma from patients or healthy control and incubated for 10 mins at room temperature. The cells were then washed using MACS buffer (0.5% BSA + 2mM EDTA in PBS) followed by staining with different clones of anti-CD38: LS198-4-2 (Beckman Coulter), JK36 (Beckman Coulter) and HIT2 (BD Biosciences) for 10 mins in the dark at room temperature. The cells were then washed and analyzed using BD LSRFortessa™ cell analyzer. Data analyses were performed using Flowjo V10.5.3 (BD).

### 2.6 Immunophenotyping by flow cytometry

PBMCs were isolated using Ficoll-Paque density gradient centrifugation, frozen in freezing medium [10% DMSO, 90% fetal bovine serum (FBS)] and stored at -80°C until use. For staining, PBMCs were thawed using complete RPMI (RPMI+10%FBS) and washed with PBS. The cells were first stained using Fixable Aqua Dead Cell Kit (Thermofisher), followed by staining with anti-CD3 (UCHT1) (Biolegend), anti-CD4 (RPA-T4) (BD Biosciences), anti-CD8 (SK1) (Biolegend), anti-CD19 (HIB19) (BD Biosciences), anti-CD27 (O323) (Biolegend), anti-CD56 (B159) (BD Biosciences), anti-CD45RA (2H4) (Beckman Coulter), anti-CD38 (HIT2) (BD Biosciences) and anti-CD38 (JK36) (Beckman Coulter). The cells were washed and analyzed using BD LSRFortessa™ cell analyzer. Data analyses were performed using Flowjo V10.5.3 (BD)

### 2.7 Immunophenotyping by mass cytometry

Frozen PBMCs were thawed using complete RPMI (RPMI+10%FBS) and washed with PBS. The cells were then treated with cisplatin for 5 mins, followed by incubation with metal-conjugated surface antibodies cocktail (**Table 1**) for 30 mins at 37°C. Cells were washed twice with CyFACS buffer (PBS with 4% FBS, 0.05% sodium azide), followed by primary antibody staining for 30 mins on ice. Subsequently, cells were washed twice with CyFACS buffer, followed by permeabilization and fixation with Foxp3 fix/perm solution (eBioscience) for 30 mins on ice. Following this, cells were washed with permeabilization buffer (Biolegend) and then stained with intracellular antibody cocktail (**Table 1**) for 30 mins on ice, wash with Biolegend permeabilization buffer then stained with metal-conjugated streptavidin for 10 mins on ice. Finally, cells were washed with PBS and fixed overnight using 2% PFA made in PBS. The next day, cells were barcoded and stained with Cell-ID Intercalator-Ir (Fluidigm) in PBS for 20 mins at room temperature. Cells were washed twice with CyFACS buffer followed by a final wash using MiliQ water and passed through size filter. Filtered cells were analyzed using Helios mass cytometer (Fluidigm) with CyTOF software version 7.0.8493. Data analyses were performed using Flowjo V10.5.3 (BD) and Cytofkit2 (33).

**Table 1:** List of antibodies used for mass cytometry staining.

	Marker	Clone	Company
Surface antibodies	CD3	UCHT1	Biolegend
	CD4	SK3	Biolegend
	CD8	SK1	Biolegend
	CD11c	B-ly6	BD Biosciences
	CD14	TüK4	Invitrogen
	CD16	3G8	Fluidigm
	CD19	HIB19	Biolegend
	CD25	M-A251	Biolegend
	CD27	LG.7F9	eBioscience
	CD38	HIT2	Biolegend
	CD39	A1	Biolegend
	CD45	HI30	Fluidigm
	CD45RA	HI100	Biolegend
	CD45RO	UCHL1	Biolegend
	CD56	NCAM16.2	BD Biosciences
	CD57	HNK-1	Biolegend
	CD127	A029D5	Biolegend
	CD161	HP-3G10	Biolegend
	CD197 (CCR7)	150503	R&D System
	HLA-DR	L243	Biolegend
	ICOS	C398.4A	Biolegend
	PD1	J116	eBioscience
Intracellular antibodies	Foxp3	PCH101	Invitrogen
	Helios	22F6	Biolegend
	Ki-67	B56	BD Pharmingen

### 2.8 Complement dependent cytotoxicity

NK92 cells were incubated with either Daratumumab, plasma from healthy control (HC) or combination therapy patient for 10 mins at room temperature. The cells were then washed using MACS buffer followed by incubating with serum from HC for 3hours at 37°C. Heat inactivated serum was included as negative control. After incubation, the cells were stained with Fixable Aqua Dead Cell Kit (Thermofisher). The cells were then washed and analyzed using BD LSRFortessa™ cell analyzer. Data analyses were performed using Flowjo V10.5.3 (BD)

## 3 Results

### 3.1 Treatment regime of the NKTL patient receiving combination therapy

**Figure 1:**
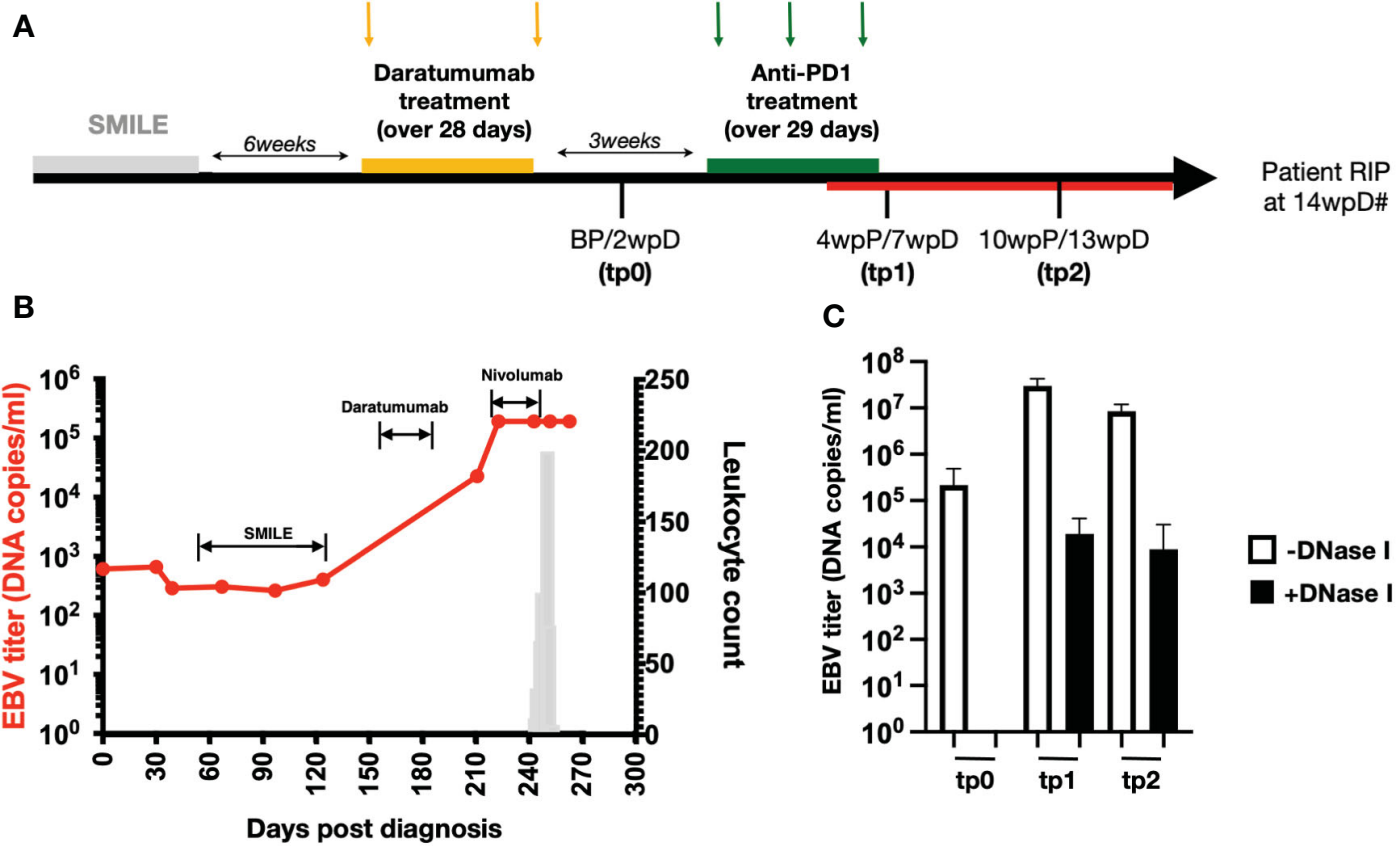
High levels of EBV load in NKTL patient who underwent combination therapy **(A)** Treatment timeline of the relapsing NKTL patient that underwent Daratumumab and anti-PD1 combination therapy (combination therapy patient). Patient was given SMILE therapy but did not respond to treatment. Six weeks after the end of SMILE therapy, patient was given 2 doses of Daratumumab over a period of 28 days. Two weeks after the last Daratumumab (2wpD) and before anti-PD1 (BP) treatment, plasma and PBMCs were collected (tp0). At 3wpD, patient was put on 3 doses of anti-PD1 (Nivolumab) treatment, spaced 15 days apart over a period of 29 days. Plasma and PBMCs were collected again at 7wpD (tp1) and 13wpD (tp2) respectively. Tp1 and tp2 also coincide with 4 weeks and 10 weeks post start of anti-PD1 (wpP) treatment. Combination therapy patient developed transient leukocytosis (indicated by red line on timeline) during anti-PD1 treatment. Hash (#) indicates demise of combination therapy patient at 14wpD. **(B)** Line graph (in red) showing plasma EBV load of the patient (left y-axis) over time after she was diagnosed with stage IVB NKTL. Labels on the chart indicate the treatment administered to the patient during that period as indicated in **(A)**. Bar chart (in grey) shows leukocyte count (right y-axis) in the patient as she develops secondary leukemia during the course of the disease. **(C)** Bar chart showing plasma EBV load in the patient at BP, tp1 and tp2 with and without DNaseI digestion prior to DNA extraction.

To study the potential outcome of combining anti-CD38 (Daratumumab) and ICI treatments, we analysed the blood samples from a relapsed/refractory patient with NKTL who was treated sequentially with Daratumumab followed by ICI (combination therapy patient). The patient did not respond to conventional SMILE (steroid, methotrexate, ifosfamide, L-asparaginase and etoposide) therapy. He was then enrolled in a Daratumumab trial (ClinicalTrials.gov ID: NCT02927925) where he received 2 cycles of 16mg/kg Daratumumab (Jassen) over a period of 4 weeks (**Figure 1A**). As NKTL cells release EBV DNA, the tumor burden could be directly associated with the amount of EBV DNA circulating in the blood (34). The increasing levels of circulating EBV-DNA levels indicated that the patient did not respond to the Daratumumab treatment (**Figure 1B**) and thus the treatment was stopped. Three weeks post Daratumumab treatment (3wpD), the patient was started on 3 cycles of off-label escalating dose of anti-PD1 (Nivolumab) (Bristol Myers Squibb). Patient was given 140mg (first cycle), 180mg (second cycle) and 200mg (third cycle), with 2 weeks interval between each cycle. However, the patient also responded poorly to this treatment (**Figure 1B**). He developed secondary leukocytosis around 7 wpD treatment before passing on at 14wpD (**Figures 1A, B**). Blood samples were collected at 2wpD (tp0), 7wpD (tp1) and 13wpD (tp2), with the latter two being taken 4 and 10 weeks post anti-PD1 (wpP), respectively (**Figure 1**).

Both NKTL patient 1 and 2 were ICI (Pembrolizumab) (Merck) treatment responder and included as reference for ICI monotherapy.

### 3.2 Increased EBV viremia and reduced levels of EBV-specific IgG

Anti-PD1 treatment did not reduce the level of circulating EBV DNA, indicating that tumor burden did not decrease (**Figures 1B, C**). Resistance to DNase digestion indicated that a fraction of the measured viral DNA was derived from virions, suggesting re-activation of the associated-virus from the lymphoma (**Figure 1C**). Analysis of the plasma EBV virus capsid antigen (VCA)-specific IgG levels further showed that the patient undergoing combination therapy had only negligible levels of EBV-specific IgG in his plasma as compared to other NKTL patients who are not treated with Daratumumab, indicating a compromised humoral anti-EBV response (**Supplementary Figure 1**).

### 3.3 Effective *in vivo* depletion of CD38+ lymphocytes by Daratumumab

**Figure 2:**
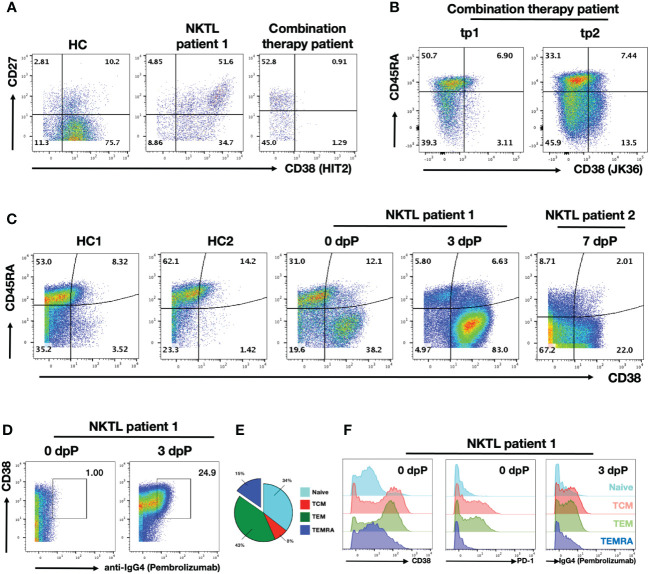
Daratumumab deplete B and activated T cells in NKTL patient undergoing combination therapy PBMC samples were obtained from healthy control (HC), NKTL patient control (NKTL patient 1) and combination therapy patient were analysed using flow cytometry. **(A)** Scatterplots showing CD27 and CD38 (HIT2 clone) expression on gated B cell subsets. Numbers in the scatterplot show the percentage of respective population within each quadrant of total B cells. **(B)** Scatterplots showing CD45RA and CD38 (JK36 clone) expressing CD8+ T cells in combination therapy patient from tp1 and tp2. Numbers in the scatterplot show the percentage of respective population within each quadrant of total CD8+ T cells. **(C)** Scatterplots showing CD45RA and CD38 (HIT2 clone) expressing CD8+ T cells in HCs and anti-PD1 treated NKTL patients (NKTL patient 1 samples obtained on 0dpP and 3dpP; NKTL patient 2 on 7dpP). Numbers in the scatterplot show the percentage of respective population within each quadrant of total CD8+ T cells. **(D)** Scatterplots showing CD38 and anti-IgG4 staining on CD8+ T cells in anti-PD1 treated NKTL patient 1 from 0dpP and 3dpP. Anti-IgG4 is used as a proxy to detect for binding of Pembrolizumab on the cells. Boxed up region shows proportion of IgG4+ cells. **(E)** Pie chart showing segregation of CD8+ T cell subsets from NKTL patient 1 into naïve (CCR7+CD45RA+), TCM (CCR7+CD45RA-), TEM (CCR7-CD45RA-) and TEMRA (CCR7-CD45RA+) based on their CCR7 and CD45RA expression prior to anti-PD1 treatment. **(F)** Histogram plots showing CD38 (left) and PD1 (middle) expression the respective on CD8+ T cells subsets in NKTL patient 1 prior to anti-PD1 treatment. Histogram plots (right) showing anti-IgG4 staining (targeting Pembrolizumab) on the respective CD8+ T cell subsets 3 days after anti-PD1 treatment.

Daratumumab is a depleting antibody known to eliminate CD38-expressing B cells (35). This subset includes plasma cells, plasmablasts and memory B cells (36). As expected, we noted a complete absence of CD38+ B cells in the patient after the Daratumumab treatment (**Figure 2A**) (12). In order to confirm the observation is not due to epitope competition between anti-CD38 staining antibody (HIT2 clone) and Daratumumab bound on the cell surface, we repeated the staining using JK36, an anti-CD38 nanobody reported not to share a common epitope with Daratumumab (**Supplementary Figure 2**) (37, 38). Flow cytometry analysis confirmed that the CD38+ B cell population is indeed missing (**Supplementary Figure 2**), which may explain the compromised humoral anti-viral response by the patient (**Supplementary Figure 1**).

While depletion of B cells and loss of antibodies is a reported side-effect of Daratumumab treatment, we also observed strong depletion of CD38+ CD8+ T cell subsets that was previously unreported (**Figure 2B**) (35). Interestingly, earlier report of Daratumumab depletion on T cell subsets was limited to only CD38+ Tregs (13). Anti-PD1 treatment aims to reinvigorate exhausted tumor-specific CD8+ T cells and this (re-)activation apparently triggers a substantial increase in the proportion of CD38+ CD8+ T cells (39, 40). We detected less than 10% of CD38+ CD8+ T cells at tp1 (7wpD/4wpP) (**Figure 2B**). The fraction of CD38+ CD8+ T cells started increasing at tp2 (10wpD/13wpP), indicating that the depleting capacity of the anti-CD38 antibody was diminishing (**Figure 2B**). Notably, the analysis of anti-PD1-treated NKTL patient revealed that the (re-)activation of T cells by anti-PD1 is associated with a strong upregulation of CD38 (**Figure 2C**). While a substantial fraction of CD38+ CD8+ T cells were already detected before the start of the therapy (day 0), the proportion of CD38+ CD8+ T cells increased substantially from 50% to 90% at day 3 after anti-PD1 treatment (Pembrolizumab) in NKTL patient 2 (**Figure 2C**). A similar activation was also observed in NKTL patient 1 at 7dpP (**Figure 2C**). Based on the CD45RA negative (CD45RA-) phenotype, these CD38+ activated cells mostly represented memory T cells (**Figure 2C**).

In order to confirm that these CD38+ T cells are re-activated by the binding of Pembrolizumab, we stained the cells using anti-IgG4. As Pembrolizumab is an IgG4 isotype, anti-IgG4 staining serves as a proxy for Pembrolizumab binding (41). Anti-IgG4 stained almost exclusively the CD38+ CD8+ T cells that were sampled 3 days after Pembrolizumab administration, confirming that anti-PD1 therapy is mediated through this subset (**Figure 2D**).

As it was previously noted that CD38+ CD8+ T cell subsets are mainly CD45RA-, we further segregated the CD8+ T cells into 4 major subsets to determine which subset Pembrolizumab is binding to. Separation of the CD8+ T cell populations into the 4 major subsets of naïve T cells (CCR7+CD45RA+), effector memory T cells (TEM, CCR7-CD45RA-), central memory T cells (TCM, CCR7+CD45RA-) and TEM expressing RA cells (TEMRA, CCR7-CD45RA+) subsets indicated that TEM and TCM comprise about half of the total CD8+ T cell population in NKTL patient 1 (**Figure 2E**) (42). Further characterization of these subsets confirmed that a large fraction TCM and TEM CD8+ T cells express CD38 (**Figure 2F**, left panel). The two subsets also expressed the highest levels of PD1 prior to the Pembrolizumab treatment indicating that these two subsets are the primary targets of ICI treatment (**Figure 2F**, middle panel). This was confirmed by the strong anti-IgG4 staining on both TCM and TEM 3 days after anti-PD1 treatment (**Figure 2F**, right panel). Notably, there were only 15% TEMRA CD8+ T cells in this patient.

### 3.4 Long-term persistence of active Daratumumab in circulation

In order to avoid potential side effects of the anti-CD38 therapy on ICI, the infusion of Daratumumab was stopped for 3 weeks prior to the start of the anti-PD1 treatment. However, it was unclear how long Daratumumab can remain functional in circulation. Earlier reports mentioned Daratumumab has a half-life of 21 days and around 11.5 ug/ml of circulating Daratumumab was detected at 6 weeks post infusion (38, 43). To determine the amount of circulating Daratumumab in the plasma of NKTL patient 1, we established an *in vitro* assay based on epitope competition. The anti-CD38 clone LS198-4-2 was found to be blocked by Daratumumab in a dose-dependent matter (**Supplementary Figure 3**). Using this antibody to stain the CD38-expressing cell line NK92 allowed us to quantify the amount of Daratumumab to concentrations as low as 500 ng/ml (**Supplementary Figure 4**). Using this assay to analyze plasma samples from NKTL patient 1, we estimated the amount of circulating Daratumumab in this patient to be 625, 25 and 2.5 ug/ml at 2 weeks (tp0), 7 weeks (tp1) and 13 wpD treatment (tp2) respectively. This is in line with what was previously reported (38, 43).

**Figure 3:**
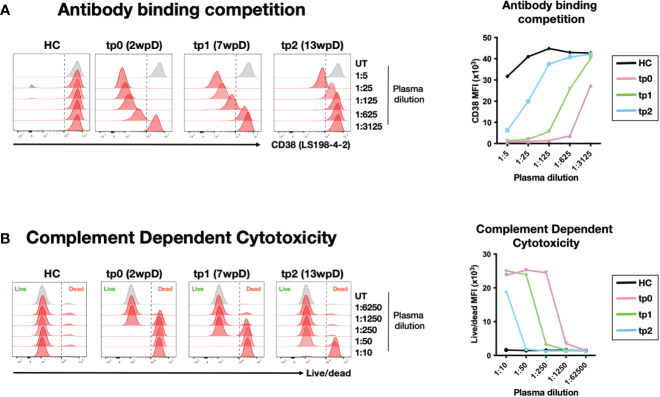
Circulating Daratumumab is functional up to 13 weeks post administration **(A)** Indirect measurement of circulating Daratumumab in combination therapy patient plasma samples. CD38-high NK92 cell line was pre-incubated with different dilution of combination therapy-treated plasma samples from tp0, tp1 and tp2 or HC. Histograms (left) and line graph showing interference of circulating Daratumumab on CD38 (clone LS198-4-2) binding using flow cytometry. Line graph (right) shows CD38 mean fluorescence intensity (MFI) on NK92 pre-incubated with combination therapy patient plasma samples. **(B)** NK92 cell line pre-treated with different dilutions of combination therapy patient plasma samples were incubated with pooled HC sera for 2-3 hours for complement dependent cytotoxicity (CDC). Cell viability was determined using live/dead staining and analysed using flow cytometry. Histogram plot (left) and line graph (right) shows live/dead MFI on NK92 after CDC was performed.

In order to determine whether circulating Daratumumab in the combination therapy patient is functional, we performed an *in vitro* complement dependent cytotoxicity (CDC) assay with NK92 target cells (44). Titration of Daratumumab showed effective CDC on NK92 at >400 ng/ml (**Supplementary Figure 4B**). The plasma samples collected at tp0, tp1 and tp2 were well above this threshold. Incubation of NK92 with these plasma samples resulted in CDC, confirming that circulating Daratumumab retains its lytic function up to 13 wpD (**Figure 3B**).

### 3.5 Anti-CD38 therapy enriches for (exhausted) PD1- TEMRA cells

**Figure 4:**
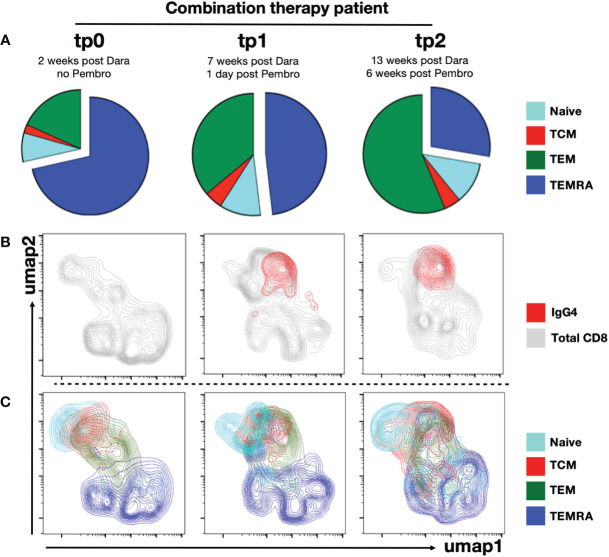
Anti-PD1 targets mainly effector and central memory CD8+ T cell subsets. PBMCs from combination therapy patient were immunophenotyped using CyTOF. CD8+ T cells were gated. **(A)** Piecharts showing distribution of into naïve, TCM, TEM and TEMRA CD8+ T cell subsets from combination therapy patient at tp0, tp1 and tp2. UMAP clustering of CD8+ T cells from combination therapy patient at tp0, tp1 and tp2 were performed. **(B)** Umap clusters of total CD8+ T cells (grey) were overlaid with anti-IgG4+ CD8+ T cells (red) or **(C)** segregated into naïve, TCM, TEM and TEMRA subsets.

Our earlier analysis on anti-PD1-treated NKTL patients suggests CD38+ TEM and TCM are most susceptible to depletion by Daratumumab treatment (**Figure 2**). In line with this, we found TCM and TEM to be strikingly underrepresented at tp0 (2wpD) in the combination therapy patient (**Figure 4A**). Almost three quarters of the CD8+ T cells from this patient were TEMRAs, which are senescent T cells with poor proliferative potential associated with poor survival in cancer patients (45).

A gradual re-emergence of TEM and proportionate reduction of TEMRA was evident at tp1 and tp2 (**Figure 4A**; **Supplementary Figure 5**). The re-emergence of TEM was also accompanied by recovery of CD38+ population at tp2 in the combination therapy patient, likely due to the waning of circulating Daratumumab at tp2 (**Figures 2B**, **3A**). This was further confirmed by e*x vivo* staining with anti-IgG4 staining, indicating that the binding of the anti-PD1 antibody (Nivolumab) was restricted to TCM and TCM, while the bulk of TEMRA cells in this patient were not targeted by the treatment (**Figures 4B, C**).

### 3.6 Anti-CD38/ICI combination therapy is ineffective in a variety of cancer types

**Figure 5:**
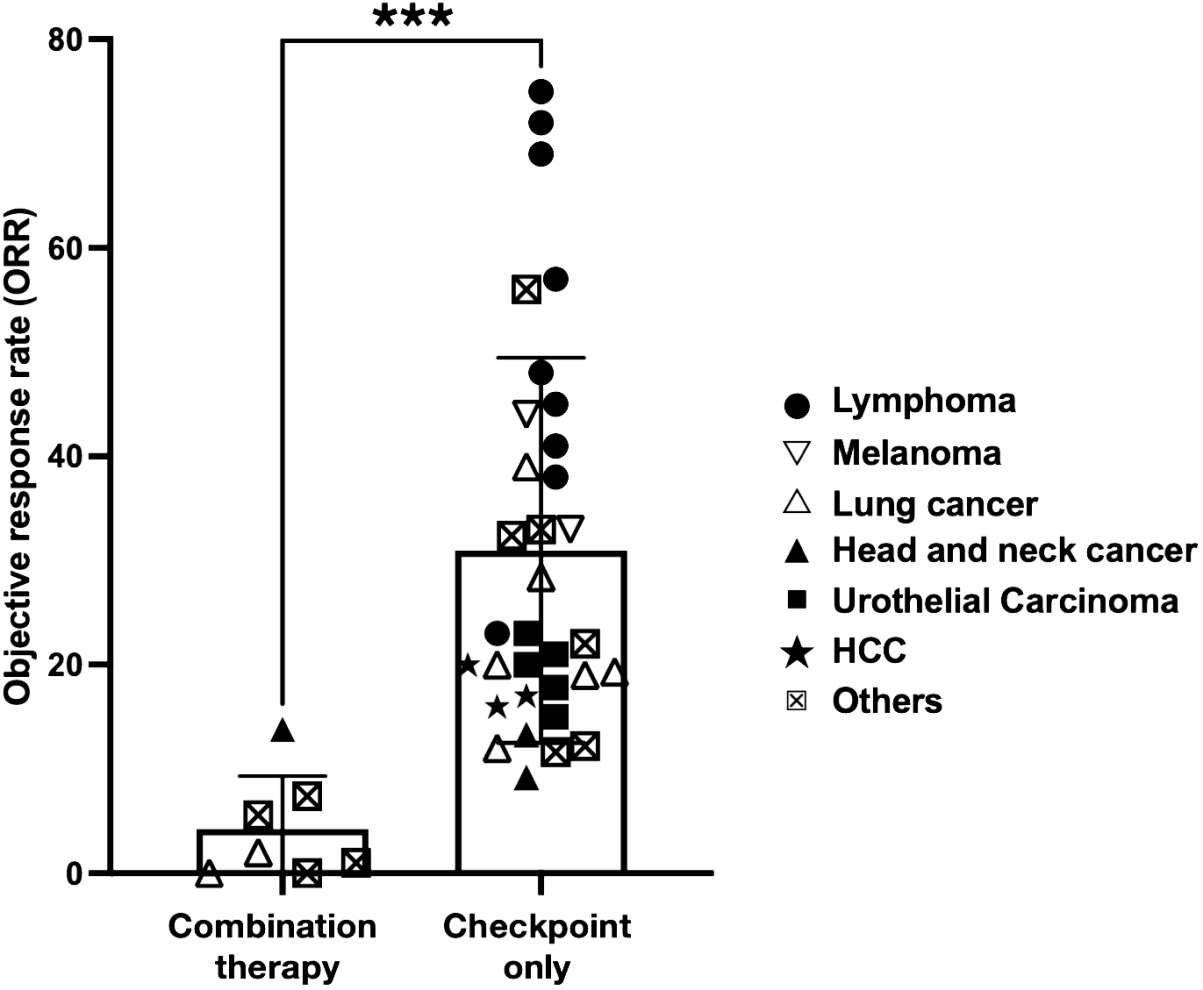
Cancer patients that received combination therapy fare significantly worse. Scatterplot comparing ORR of clinical trials performed in cancer patients that received combination therapy (anti-CD38 and immune checkpoint inhibitor (ICI) or ICI monotherapy only. ICI includes either anti-PD1 or anti-PDL1 therapy. The symbols indicate type of cancer that is being treated in the trial. Statistical analysis was performed using two-tailed Mann-Whitney U test. A p-value of less than 0.05 is considered statistically significant. (***p<0.0001).

To assess the efficacy of combining anti-CD38 and PD1/PDL1-directed ICI in more generalizable scenario, we did a comprehensive literature search and compared the objective response rate (ORR) of small scale- and clinical trial-studies that administer either a combination of anti-CD38/ICI or ICI alone in various forms of cancer (**Table 2**). Taking into account all the published data of these studies, we noted a significant reduction in the ORR of patients undergoing combination (46, 62, 66) therapy as compared to ICI alone (24, 25, 27, 46–61, 63–65, 67–76) (**Figure 5**). This was independent of the type of PD1/PDL1 interference, as no significant difference in ORR was observed whether anti-PD1 or anti-PDL1 monotherapy was applied (**Supplementary Figure 7**). In our case study as well as these published combination therapies, anti-CD38 was given prior to the application of the PD1-based ICI. Thus, a combination of these two therapies in this order seem to be counter-productive (46, 62, 66).

**Table 2:** List of anti-CD38 and/or checkpoint inhibitor clinical trials.

Authors	Cancer type	n	Treatment type	Anti-PDL1	Anti-PD1	Anti-CD38	ORR (%)	Phase	Age (Range)	Male (%)	Reference
Armand et al	B cell lymphoma	21	Checkpoint		Pembrolizumab	–	48	Ib	31 (22-62)	33	(47)
Armand et al	B cell lymphoma	53	Checkpoint	–	Pembrolizumab	–	45	II	33 (20-61)	43	(47)
Chung et al	Cervical cancer	98	Checkpoint	–	Pembrolizumab	–	12.2	II	46 (24-75)	33	(48)
Overman et al	Colorectal cancer	74	Checkpoint	–	Nivolumab	–	32.4	II	52·5 (44–64)	59	(49)
Fuchs et al	Gastroesophageal cancer	259	Checkpoint	–	Pembrolizumab	–	11.6	II	62.0 (24-89)	76.4	(50)
Simonelli et al	Glioblastoma	33	Combination	Atezolizumab		Isatuximab	0	I/II	55.0 (21-75)	69.7	(62)
Simonelli et al	Hepatocellular carcinoma	27	Combination	Atezolizumab		Isatuximab	7.4	I/II	62.8 (42-82)	74.1	(62)
Zhu et al	Hepatocellular carcinoma	104	Checkpoint	–	Pembrolizumab	–	17	II	68 (62–73)	83	(51)
Verset et al	Hepatocellular carcinoma	51	Checkpoint	–	Pembrolizumab	–	16	II	68 (41-91)	86	(52)
El-Khoueiry et al	Hepatocellular carcinoma	214	Checkpoint	–	Nivolumab	–	20	I/II	64 (55-70)	79	(53)
Simonelli et al	Head and neck cancer	29	Combination	Atezolizumab		Isatuximab	13.8	I/II	62.0 (40-76)	89.7	(62)
Ferris et al	Head and neck cancer	240	Checkpoint	–	Nivolumab	–	13.3	III	59 (29-83)	82.1	(54)
Guigay et al	Head and neck cancer	153	Checkpoint	Avelumab	–	–	9.2	I	63 (37–91)	81.7	(55)
Chen et al	Hodgkin Lymphoma	210	Checkpoint	–	Pembrolizumab	–	69	II	35 (18-76)	53.8	(56)
Wolchok et al	Melanoma	316	Checkpoint	–	Nivolumab	–	44	III	60 (25-90)	64	(57)
Robert et al	Melanoma	556	Checkpoint	–	Pembrolizumab	–	33	III	62 (51-71)	60.3	(58)
Nghiem et al	Merkel Cell carcinoma	50	Checkpoint	–	Pembrolizumab	–	56	II	70.5 (46-91)	68	(59)
Kaufman et al	Merkel Cell carcinoma	88	Checkpoint	Avelumab	–	–	33	II	72.5 (64.5-77)	74	(60)
Huang et al	NKTL	32	anti-CD38	–	–	Daratumumab	25	II	56.0 (22–78)	71.9	(63)
Kwong et al	NKTL	7	Checkpoint	–	Pembrolizumab	–	72	–	49 (31-68)	100	(25)
Li et al	NKTL	7	Checkpoint	–	Pembrolizumab	–	57	–	47 (17-61)	57	(24)
Tao et al	NKTL	28	Checkpoint	–	Sintilimab	–	75	II	37 (19-65)	67	(61)
Huang et al	NKTL	29	Checkpoint	CS1001	–	–	41	II	44 (30-74)	55.2	(63)
Kim et al	NKTL	21	Checkpoint	Avelumab	–	–	38	II	54 (24-78)	62	(27)
Kim et al	Non-Hodgkin lymphoma	30	Checkpoint	–	Pembrolizumab	–	23	–	49 (18-80)	70	(64)
Zucali et al	Non-small cell lung carcinoma	20	Combination	–	Cemiplimab	Isatuximab	0	I/II	65.5 (53–77)	0	(66)
Mok et al	Non-small cell lung carcinoma	637	Checkpoint	–	Pembrolizumab	–	39	III	63 (56-69)	71	(67)
Vokes et al	Non-small cell lung carcinoma	135	Checkpoint	–	Nivolumab	–	20	III	62 (39-85)	82	(68)
Vokes et al	Non-small cell lung carcinoma	292	Checkpoint	–	Nivolumab	–	19	III	61 (37-84)	52	(68)
Antonia et al	Non-small cell lung carcinoma	473	Checkpoint	Durvalumab	–	–	28.4	III	64 (31-84)	70.2	(76)
Pillai et al	Non-small cell lung carcinoma	46	Combination	Atezolizumab	–	Daratumumab	4.3	Ib/II	65.5 (38–85)	82.6	(46)
Pillai et al	Non-small cell lung carcinoma	46	Checkpoint	Atezolizumab	–	–	13	Ib/II	61.0 (30–81)	60.9	(46)
Simonelli et al	Ovarian cancer	18	Combination	Atezolizumab		Isatuximab	5.6	I/II	55.0 (35-80)	0	(62)
Zucali et al.	Prostate cancer	24	Combination	–	Cemiplimab	Isatuximab	4.2	I/II	69.5 (61–88)	100	(66)
Chung et al	Small cell lung carcinoma	83	Checkpoint	–	Pembrolizumab	–	19.3	Ib/II	62 (24-84)	63.9	(69)
Ready et al	Small cell lung carcinoma	147	Checkpoint	–	Nivolumab	–	12	I/II	63 (29-83)	58.5	(70)
Kojima et al	Squamous cell carcinoma	314	Checkpoint	–	Pembrolizumab	–	22	III	63 (23-84)	86.9	(71)
Bellmunt et al	Urothelial Carcinoma	542	Checkpoint	–	Pembrolizumab	–	21	III	67 (29-88)	74.1	(72)
Galsky et al	Urothelial Carcinoma	270	Checkpoint	–	Nivolumab	–	20	II	65.0 (38–90)	78.1	(73)
Rosenberg	Urothelial Carcinoma	310	Checkpoint	Atezolizumab	–	–	15	II	66 (32–91)	78	(74)
Balar et al	Urothelial Carcinoma	119	Checkpoint	Atezolizumab	–	–	23	II	73 (51-92)	81	(65)
Powles et al	Urothelial Carcinoma	191	Checkpoint	Durvalumab	–	–	17.8	I/II	67.0 (34-88)	71.2	(75)

## 4 Discussion

NKTL patients that failed L-asparaginase chemotherapy are usually faced with a dismal outcome (77). While ICI has shown to be a promising treatment for relapsing NKTL patients, the ORR ranges from 37-54% (11, 24, 25, 27, 61). As ICI and anti-CD38 therapy function through different mechanisms, the possibility of combining ICI and anti-CD38 to improve patient outcome was raised (30). Here we present data from a relapsed NKTL patient who underwent combination therapy to demonstrate that the combined use of anti-CD38 and anti-PD1 produced antithetic effects. This was confirmed in a meta-study based on data from published cancer trials showing that ORR from combination therapies is significantly reduced as compared to ICI monotherapies.

ICI monotherapy is reportedly effective in a subset of refractory NKTL patients (24–27). As shown in the anti-PD1 treated NKTL patients, anti-PD1 treatment triggered a strong activation and expansion of the CD45RA- memory cells TCM and TEM. The two subsets expressed the highest levels of PD1 resulting in a strong binding of the anti-PD1 antibody (Pembrolizumab). As this reactivation is associated with an upregulation of CD38, these subsets were depleted by Daratumumab, leaving mostly TEMRA cells as the predominant CD8+ T cell subset in patient. TEMRAs are defined as terminally differentiated senescent memory cells with low replicative potential and typically are associated with poor anti-tumor response (42, 78).

Daratumumab is used for treating multiple myeloma (MM), a malignancy arising from CD38 high plasma cells (9, 12). It is thus unsurprising to find the patient to be severely depleted of CD38+ B cells such as plasma cells (35). Depletion of these cells likely accounted for the low anti-EBV IgG titer. The loss of antibody titer and B cell responses can also cause patients to be more susceptible to infections (79), which could be a contributing factor to the uncontrollable increase in EBV viremia and small burst of virion production as evidenced by the detection of encapsidated viral DNA.

On top of this, a more important factor associated with the failure of the combination therapy is likely the depletion of (reactivated) CD38+ T cells. Upregulation of CD38+ on activated CD8+ T cells has been widely used as a prognostic marker for various diseases, including HIV infection (15, 17) and chronic lymphocytic leukemia (80). The importance of these CD38+ leukocytes was observed in hepatocarcinoma patients, where patients with higher levels of infiltrating CD38+ cells were found to respond better to anti-PD1 treatment (81). A recent study in non-small cell lung carcinoma (NSCLC) patients also showed that a higher level of tumor-infiltrating CD38+ CD8+ T cells is correlated with better survival outcomes. In line with our own observation, their data further suggest that CD38+ CD8+ T cells are the prime target for ICI reinvigoration (82) and that CD38 was upregulated on CD8+ T cells after anti-PD1 therapy (40, 83). In agreement with these data, we also noted that Pembrolizumab was predominantly detected on CD38-expressing CD8+ T cells.

Daratumumab shows potent depleting effect. At 7 wpD treatment, we found only negligible level of CD38+ CD8+ T cells in the patient blood. High levels of circulating Daratumumab were shown to be fully functional up to 13wpD through *in vitro* CDC assay, suggesting that any activated CD38+ CD8+ T cells induced by the anti-PD1 treatment are likely being depleted by the circulating Daratumumab. This notion is supported by the re-emergence of CD38+ CD8+ T cells coinciding with waning levels of circulating Daratumumab at 13wpD. Further analysis of the re-emerging CD38+ CD8+ subsets revealed that these are CD45RA- TCM and TEM populations, the same subsets expressing PD1 and hence are targeted by anti-PD1 therapy. While the depletion of NK cells (13, 14) and B cells has been previously reported, activated CD8+ T cells are also apparently effectively removed by this antibody.

Taken together, our results show that Daratumumab can persist in the circulation for up to 13 weeks without losing its lytic function. As the activation of CD8+ T cells by the anti-PD1 treatment results in the upregulation of CD38, these reinvigorated cells become an immediate target for Daratumumab-mediated lysis, thus abrogating the effectiveness of anti-PD1 treatment. Notably, clinical trial involving the use of Daratumumab and Atezolizumab (anti-PDL1) (NCT03023423) was also halted due to the lack of clinical efficacy (46).

## 5 Conclusions

Despite the small sample size, this study has important clinical implications in the treatment of NKTL patients. Our results provided strong evidence to caution against the combined use of anti-PD1 and anti-CD38 agents, especially in the sequential order of anti-CD38 followed by anti-PD1.

## Data availability statement

The original contributions presented in the study are included in the article/**Supplementary Material**. Further inquiries can be directed to the corresponding authors.

## Ethics statement

Healthy donor samples were collected under the SingHealth Centralised Institutional Review Board (CIRB Ref: 2017/2806). Patient samples were obtained under SingHealth CIRB Ref: 2004/407/F. Written informed consent was obtained from all donors prior to sample collection. Written informed consent was obtained from the individual(s) for the publication of any potentially identifiable images or data included in this article.

## Author contributions

WL: Writing – review & editing, Writing – original draft, Validation, Supervision, Project administration, Methodology, Investigation, Formal Analysis, Data curation, Conceptualization. JQL: Writing – review & editing, Writing – original draft, Data curation. TT: Writing – review & editing, Resources, Conceptualization. DT: Writing – review & editing, Resources, Methodology. SK: Writing – review & editing, Writing – original draft, Project administration, Investigation, Data curation. KP: Writing – review & editing, Writing – original draft, Supervision, Project administration, Methodology. LW: Writing – review & editing, Writing – original draft, Supervision, Project administration, Methodology. JL: Writing – review & editing, Writing – original draft, Supervision, Project administration, Methodology. KT: Writing – review & editing, Writing – original draft, Validation, Project administration, Methodology. WC: Writing – review & editing, Validation, Supervision. SL: Writing – review & editing, Resources, Funding acquisition. CO: Writing – review & editing, Resources, Methodology, Investigation, Funding acquisition, Data curation. OR: Writing – review & editing, Writing – original draft, Supervision, Resources, Funding acquisition, Formal Analysis, Conceptualization.
