# Supplementary figures and images for: Counterproductive effects of anti-CD38 and checkpoint inhibitor for the treatment of NK/T cell lymphoma
Source: Front Immunol. 2024 Apr 12;15:1346178. doi: 10.3389/fimmu.2024.1346178 (PMC11045949; doi:10.3389/fimmu.2024.1346178)

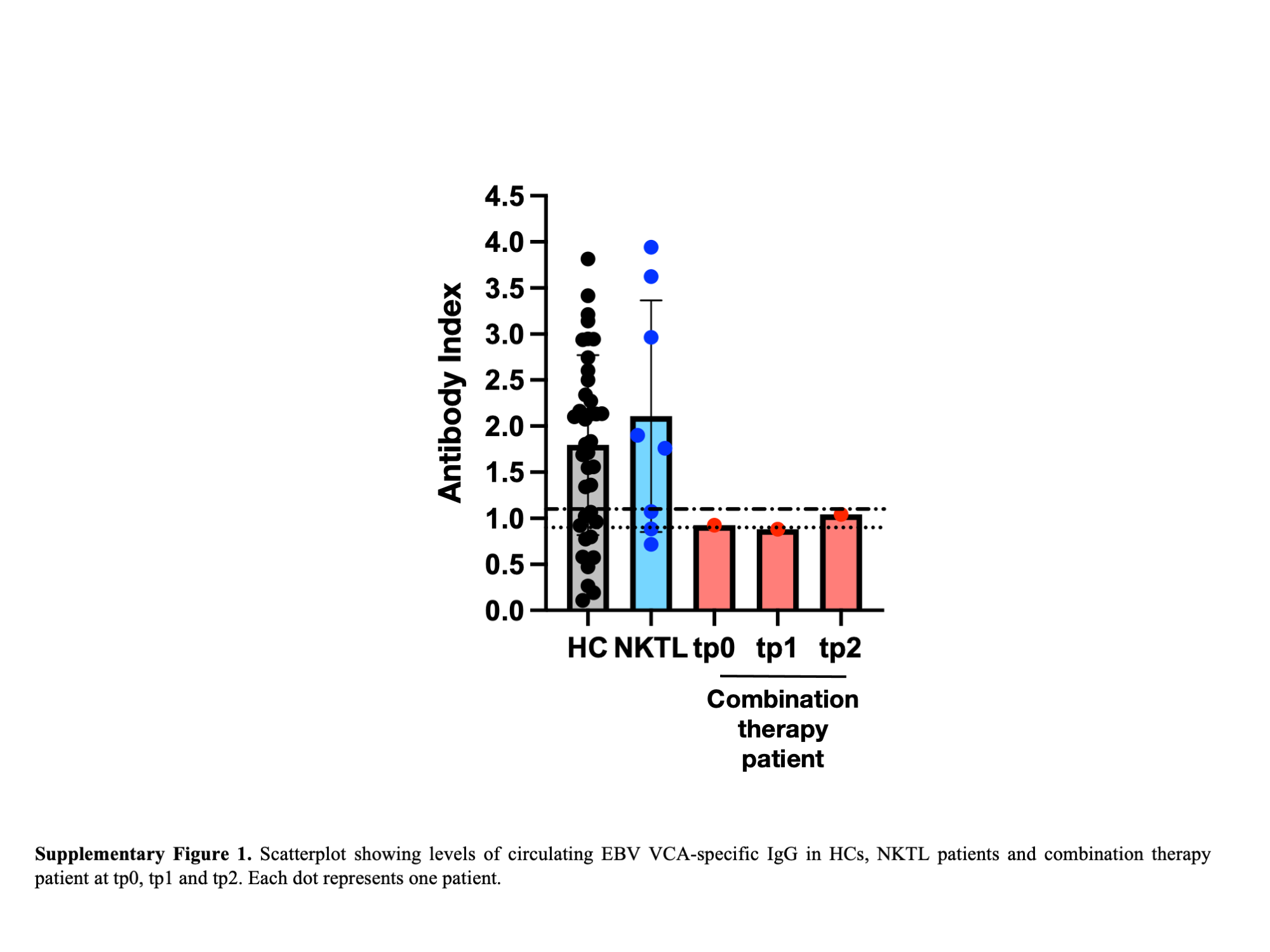

Supplement: Supplementary file 1 [file Image_1.tiff]

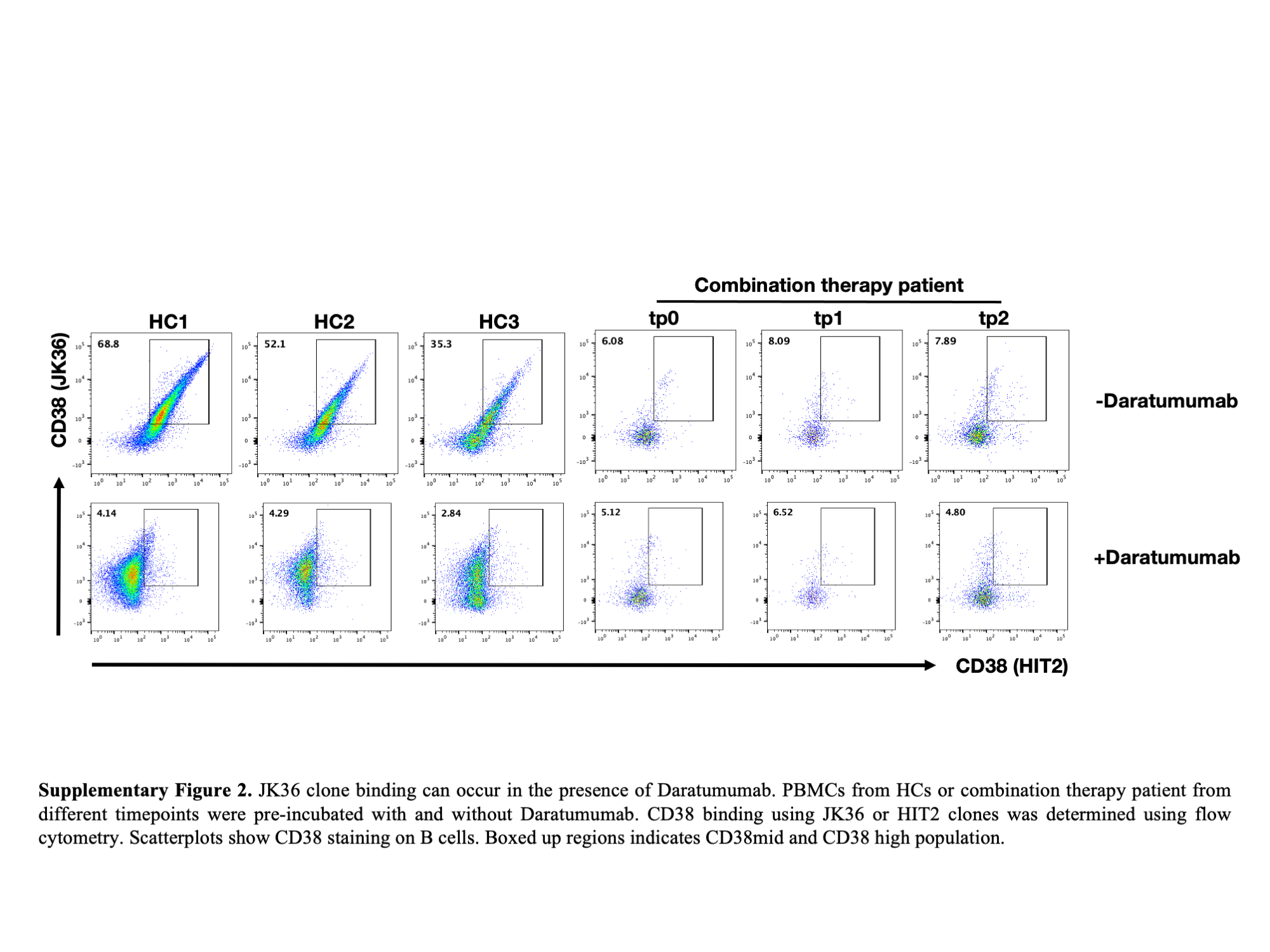

Supplement: Supplementary file 2 [file Image_2.tiff]

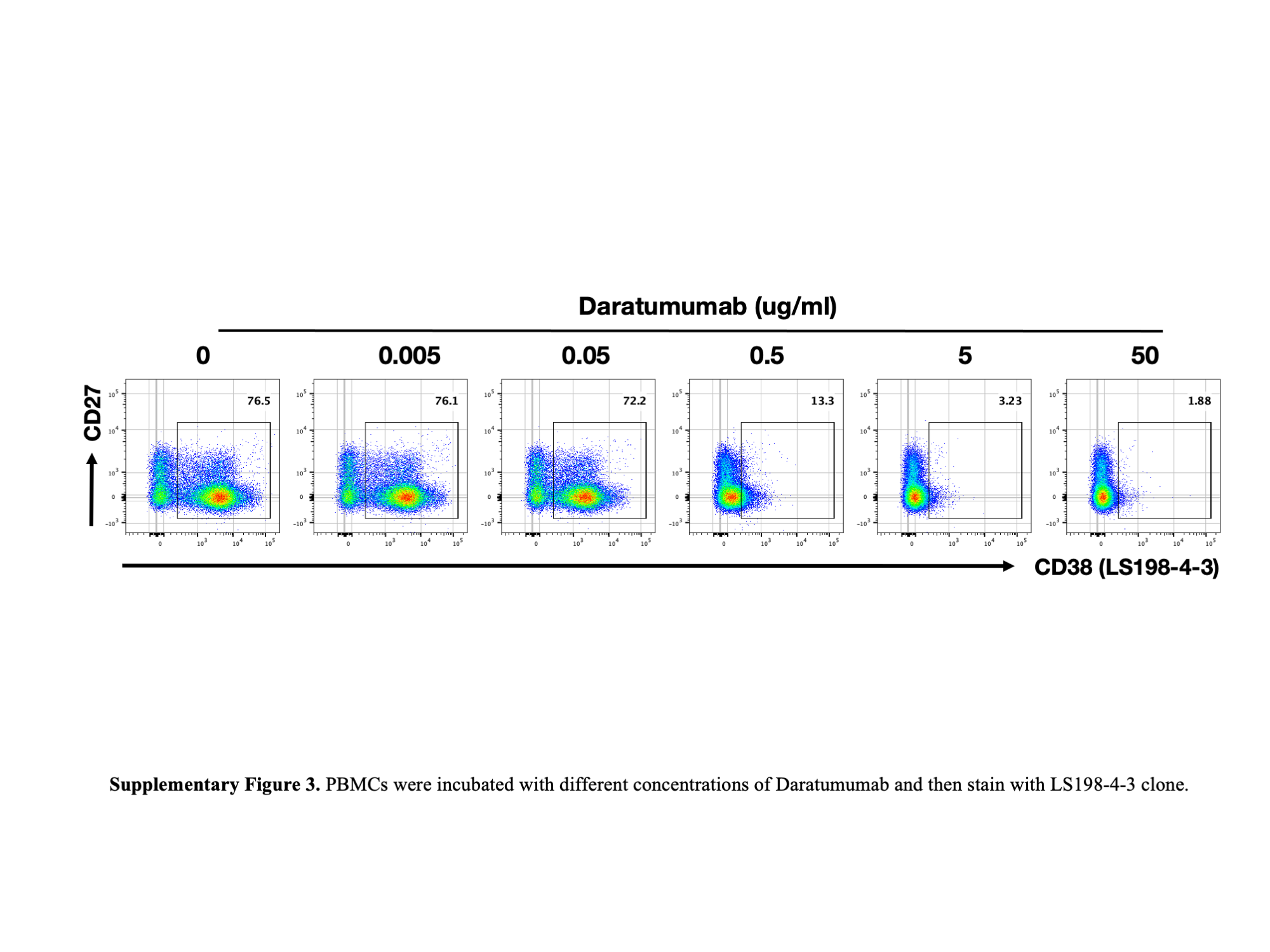

Supplement: Supplementary file 3 [file Image_3.tiff]

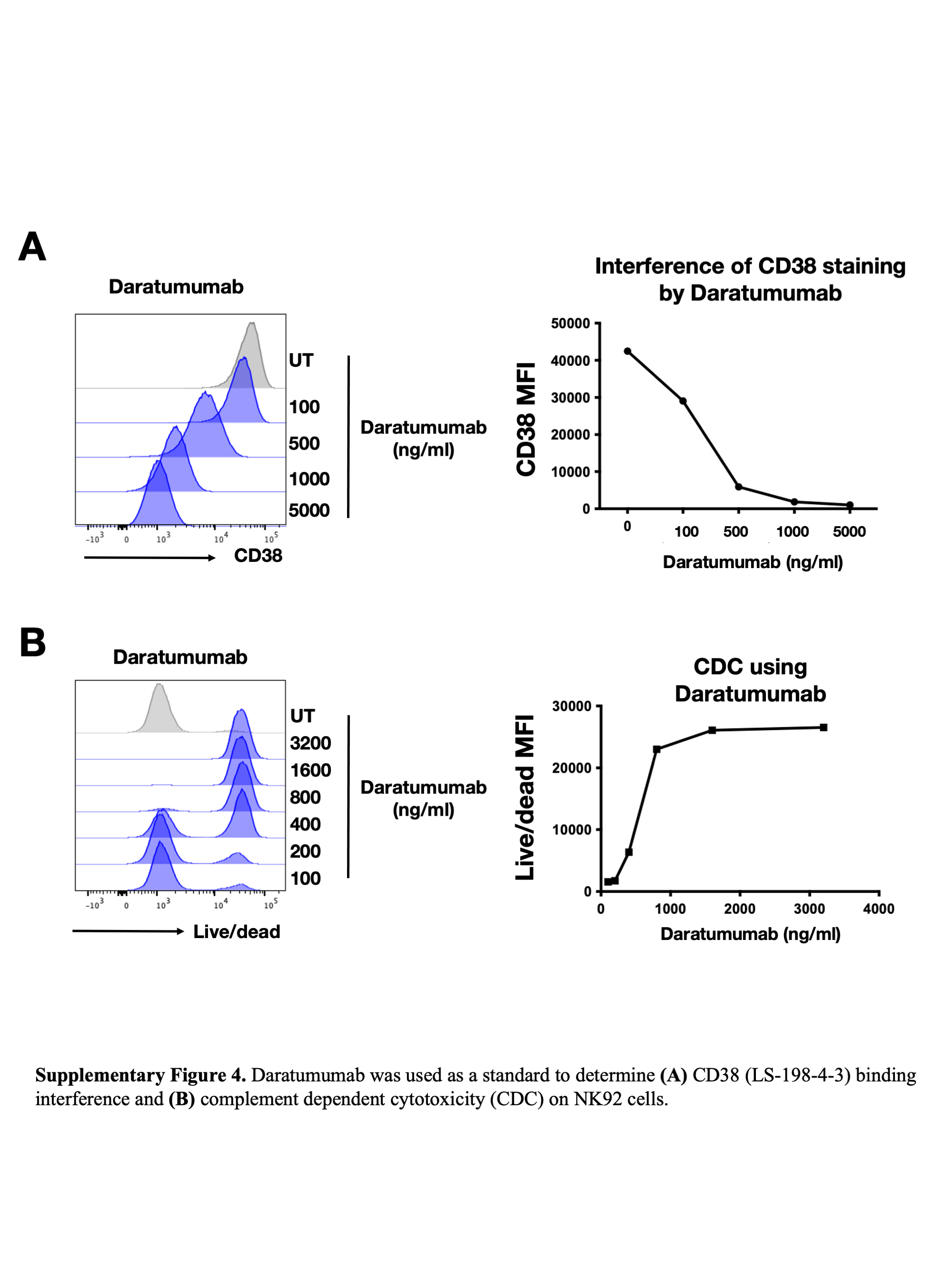

Supplement: Supplementary file 4 [file Image_4.tiff]

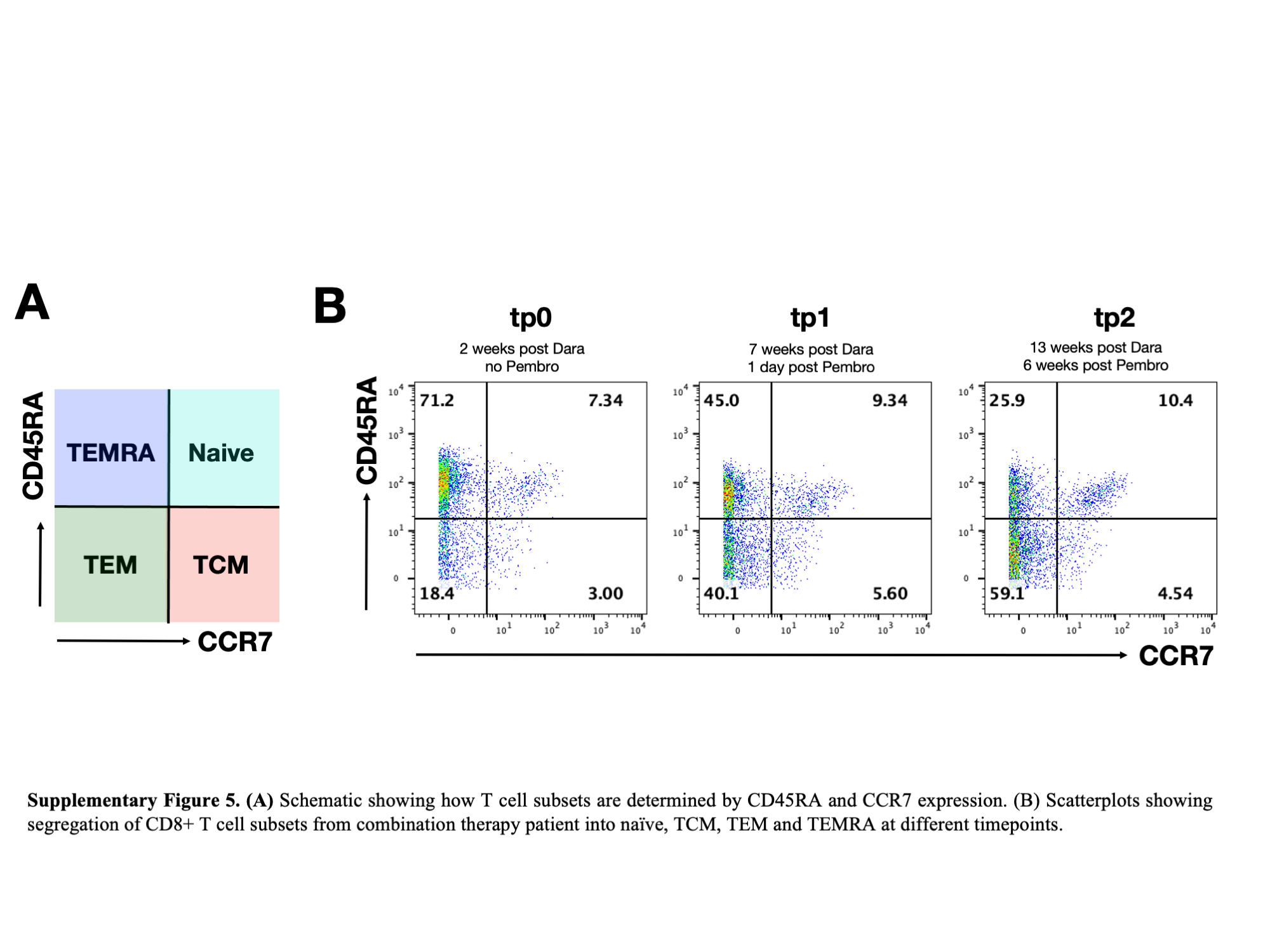

Supplement: Supplementary file 5 [file Image_5.tiff]

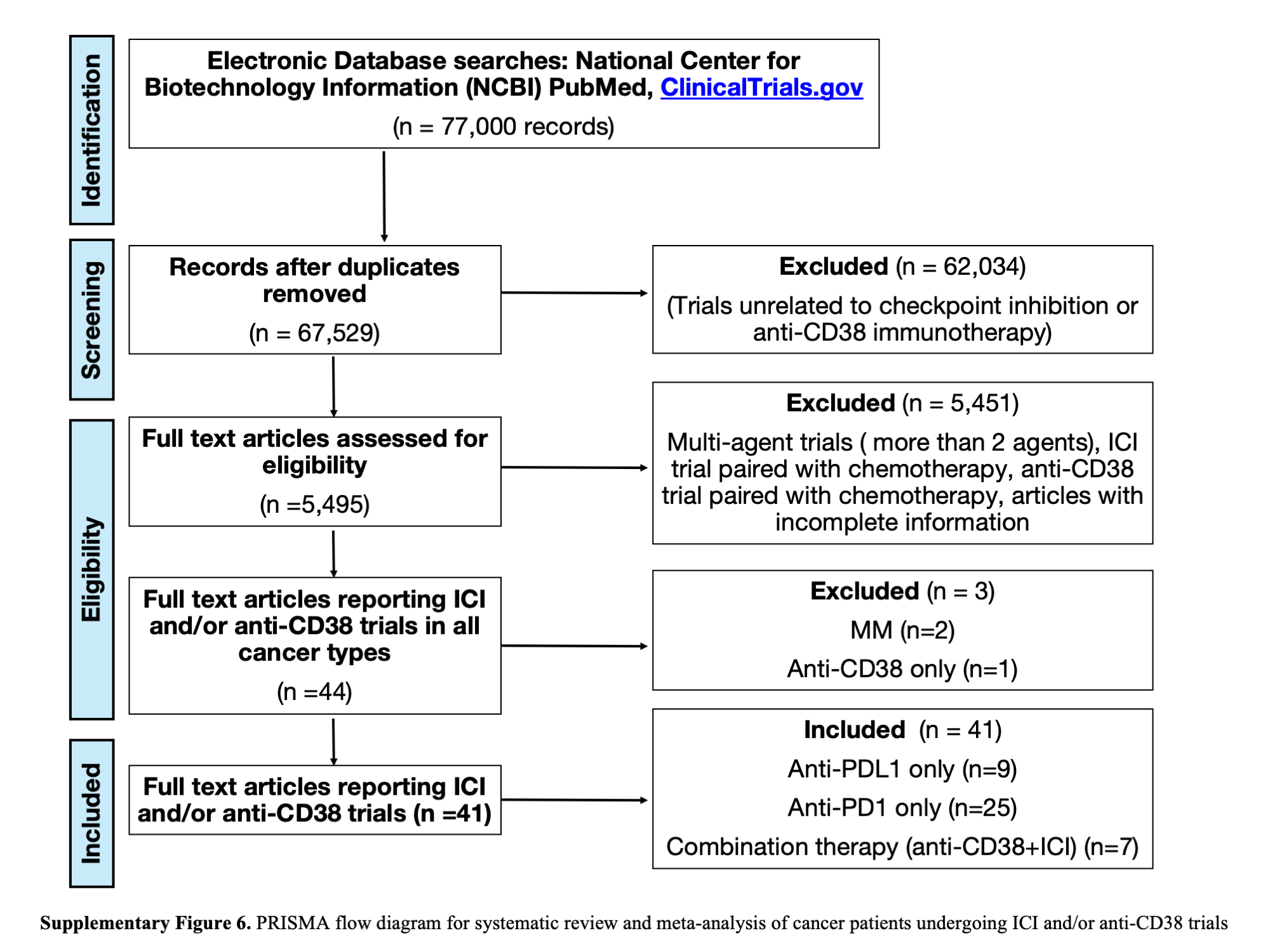

Supplement: Supplementary file 6 [file Image_6.tiff]

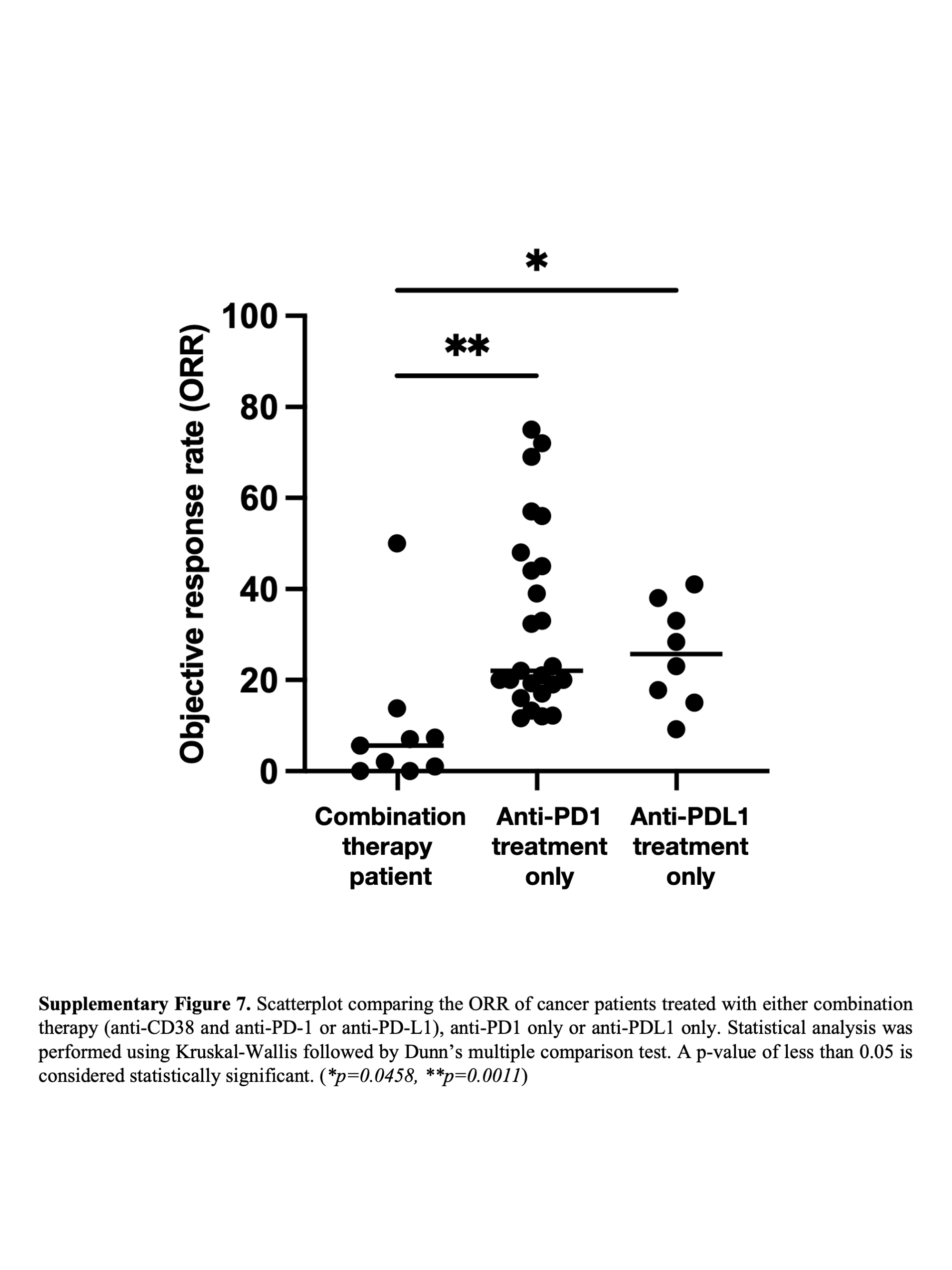

Supplement: Supplementary file 7 [file Image_7.tiff]
